# Increasing caesarean section rates among low-risk groups: a panel study classifying deliveries according to Robson at a university hospital in Tanzania

**DOI:** 10.1186/1471-2393-13-107

**Published:** 2013-05-08

**Authors:** Helena Litorp, Hussein L Kidanto, Lennarth Nystrom, Elisabeth Darj, Birgitta Essén

**Affiliations:** 1 Department of Women’s and Children’s Health, Uppsala University, 751 85, Uppsala, Sweden; 2 Department of Obstetrics and Gynaecology, Muhimbili National Hospital, Kallenga street, Upanga, PO Box 65439, Dar es Salaam, Tanzania; 3 Department of Public Health and Clinical Medicine, Epidemiology and Global Health, Umeå University, 901 85, Umeå, Sweden

**Keywords:** Caesarean section, Robson classification, Low-income countries

## Abstract

**Background:**

Rising caesarean section (CS) rates have been observed worldwide in recent decades. This study sought to analyse trends in CS rates and outcomes among a variety of obstetric groups at a university hospital in a low-income country.

**Methods:**

We conducted a hospital-based panel study at Muhimbili National Hospital, Dar es Salaam, Tanzania. All deliveries between 2000 and 2011 with gestational age ≥ 28 weeks were included in the study. The 12 years were divided into four periods: 2000 to 2002, 2003 to 2005, 2006 to 2008, and 2009 to 2011. Main outcome measures included CS rate, relative size of obstetric groups, contribution to overall CS rate, perinatal mortality ratio, neonatal distress, and maternal mortality ratio. Time trends were analysed within the ten Robson groups, based on maternal and obstetric characteristics. We applied the *χ*^2^ test for trend to determine whether changes were statistically significant. Odds ratios of CS were evaluated using multivariate logistic regression, accounting for maternal age, referral status, and private healthcare insurance.

**Results:**

We included 137,094 deliveries. The total CS rate rose from 19% to 49%, involving nine out of ten groups. Multipara without previous CS with single, cephalic pregnancies in spontaneous labour had a CS rate of 33% in 2009 to 2011. Adjusted analysis explained some of the increase. Perinatal mortality and neonatal distress decreased in multiple pregnancies (*p* < 0.001 and *p* = 0.003) and nullipara with breech pregnancies (*p* < 0.001 and *p* = 0.024). Although not statistically significant, there was an increase in perinatal mortality (*p* = 0.381) and neonatal distress (*p* = 0.171) among multipara with single cephalic pregnancies in spontaneous labour. The maternal mortality ratio increased from 463/100, 000 live births in 2000 to 2002 to 650/100, 000 live births in 2009 to 2011 (*p* = 0.031).

**Conclusion:**

The high CS rate among low-risk groups suggests that many CSs might have been performed on questionable indications. Such a trend may result in even higher CS rates in the future. While CS can improve perinatal outcomes, it does not necessarily do so if performed routinely in low-risk groups.

## Background

In recent decades, rising caesarean section (CS) rates have been observed worldwide [1]. There are many reasons this trend is considered a problem. CS is associated with increased risk of blood transfusion, hysterectomy and death as compared to vaginal delivery [2] and a uterine scar can increase the risk of uterine rupture, placenta accreta and placenta previa in a subsequent pregnancy [3,4]. Since CS entails higher costs than vaginal delivery [5], CSs done routinely without medical indication could represent a drain on resources and have negative implications for health equity [6]. Previous studies have explored rising CS rates in high-income [7] and in middle to lower-middle income countries [8-10]. While maternal request [11], electronic fetal heart rate monitoring [12], and defensive obstetric practices [13] have been proposed as reasons for elevated CS rates in high- and middle-income countries, explanations for elevated CS rates in low-income countries are likely to be different. To our knowledge, few previous studies have explored a high CS rate in a low-income country [14-16].

Since its publication in 2001, the Robson ten-group classification has been accepted as a means of analysing and comparing CS rates throughout the world [17]. While indications for CS may vary according to the obstetrician making the decision to perform the procedure, the Robson classification objectively assesses the CS rate among women with different obstetric characteristics. The classification has been applied in several studies conducted in high- and middle-income countries [18,19]. Although using the system requires a minimum of resources, to our knowledge only one previous study has applied the Robson classification to a low-income country [20]. Our aim was to analyse trends in CS rates and perinatal and maternal outcomes among different obstetric groups in a low-income country using the Robson classification.

## Methods

### Design and source of data

We conducted a panel study of all deliveries between 2000 and 2011 extracted from the obstetric database at Muhimbili National Hospital (MNH), Tanzania. The database, which has been described in detail elsewhere [21], prospectively collects information on patient characteristics, antenatal care, labour, and maternal and neonatal outcomes of all deliveries of gestational age ≥ 28 weeks at MNH. Outcomes of newborns admitted to the neonatal unit are recorded in the obstetric database for a period up to seven days after delivery. All deliveries with complete information on selected variables were included in the study. Exclusion criteria were incomplete information on year, parity, plurality, birth weight, or mode of delivery, because missing information in these variables would prevent classification into the Robson groups. Deliveries with missing information on perinatal mortality (alive/dead), Apgar score at five minutes, or maternal mortality (alive/dead) were also excluded. Incomplete information in other variables was interpreted as described in the paragraphs below.

### Study setting

MNH is situated in Dar es Salaam, the largest city in Tanzania, with an estimated four million inhabitants. Tanzania is a low-income country that has a total fertility rate of 5.4 children per woman and a national CS rate of 5.0% [22]. It is the policy of the government that maternal care and medical services for children under five years of age are provided free of charge. The current referral system has a pyramidal pattern: patients are referred from dispensaries and health centres to district and regional hospitals. In the Dar es Salaam area, most people live within ten kilometres of a health care facility, and 90% of all deliveries are attended by a skilled professional [22].

MNH is the largest public hospital in Tanzania and serves as a teaching and referral institution. One specialist obstetrician, one consultant obstetrician and two residents are on call every day. Either the specialist obstetrician or the consultant obstetrician is usually involved in the CS decision-making process. About three-quarters of the women who give birth at MNH are self-referred. Since 2004, the obstetric department runs as a public/private partnership where women with private healthcare insurance are given separate rooms and are attended by a specialist responsible for their delivery. When performing CS on a private patient, doctors receive extra compensation from the patient’s healthcare insurance. In addition to self-referred women, obstetric patients come from the entire Dar es Salaam region, including three public district hospitals (Temeke, Amana, and Mwanamayala) and Lugalo military hospital. During the mid-2000s, all hospitals in the city underwent extensive renovation, resulting in an increase in the number of deliveries at district hospitals and a decrease in deliveries at MNH [23]. Although district hospitals are now capable of performing CS when needed, their CS rates remain low (between 5% and 8% according to hospital registers) due to constant overcrowding of birthing women and a lack of supplies and staff. At MNH, on the other hand, the CS rate rose from 16% to 27% between 1999 and 2005 [14].

### The Robson classification system

The Robson classification provides a framework for monitoring and auditing CS rates. It is based on four obstetric concepts: category of pregnancy, previous obstetric history, course of pregnancy, and gestational age. On this basis women are categorised into ten groups [24]. The classification process is mutually exclusive and all inclusive, which means that every woman fits into one group, and one group only.

Applying the Robson classification to the obstetric database at MNH, we categorised women into ten groups on the basis of parity (nullipara/multipara), previous CS (previous CS/no previous CS), plurality (single/multiple), presentation (cephalic/breech/transverse), labour (spontaneous/induced/no labour), birth weight (< 2.5 kg or ≥ 2.5 kg), and mode of delivery (CS/no CS). For each group, the CS rate (number of CSs/total number of deliveries), relative size (total deliveries in each group/total deliveries), and absolute contribution to the total CS rate (CS deliveries in each group/total deliveries) were calculated.

### Definition of variables

Variables collected to characterize the population were maternal age, parity, private health care insurance, referral status (referred by another hospital/self-referred), and use of vacuum extraction. We classified as “public” all women admitted before 2004, when the system of private healthcare insurance was introduced. Women with unknown referral status (n = 188) were considered self-referred.

For some variables, information in the database had to be interpreted or modified in order to apply the Robson classification. For example, women were considered to have had a previous CS if this was indicated on their antenatal card, diagnosed in hospital, or cited as an indication for repeat CS. If not stated among these variables, a woman was considered not to have had a previous CS. No variable specifically described presentation; thus, all deliveries without a diagnosis of breech or transverse lie were considered cephalic. In the same manner, all deliveries not labelled as induced were considered spontaneous labour, even if data were lacking. Because the database underwent continuous development during the study period, the definition of gestational age had to be modified. The variable gestational age was based on fundal height from 2000 to 2004, and on the last menstruation period from 2005 to 2011. To avoid this source of error from affecting the results, we used a birth weight of < 2.5 kg to estimate gestational age of < 37 weeks [25].

Perinatal mortality ratio, neonatal distress, and maternal mortality ratio were chosen to characterise perinatal and maternal outcomes. Perinatal mortality ratio was defined as the total number of stillbirths and early neonatal deaths occurring at hospital within seven days after birth per 1000 deliveries. Neonatal distress was described as the number of live births with an Apgar score < 7 at five minutes per total number of live births. Outcomes for twin deliveries were calculated for the first twin only, since the study aimed to evaluate the trend rather than the outcome. Perinatal outcomes were analysed separately in the ten Robson groups. To estimate maternal outcomes, we calculated the maternal mortality ratio (number of maternal deaths per 100,000 live births). Since maternal deaths are rare, they were not analysed within the ten Robson groups, but presented as a total.

### Statistical analysis

Data were captured by Epi Info and exported to SPSS version 20 for statistical analysis. To allow a comprehensive analysis of time trends, the 12 years were divided into four periods: 2000 to 2002, 2003 to 2005, 2006 to 2008, and 2009 to 2011. One-way ANOVA was applied to detect temporal changes in mean maternal age and mean parity, as these were considered important variables for understanding the time trend of the CS rate. The *χ*^2^ test for trend was used to detect temporal changes in CS rate, relative size of obstetric groups, perinatal mortality ratio, neonatal distress, and maternal mortality ratio. The relative change in CS rate between the first and last time period was calculated by dividing the change in CS rate with the CS rate for the first time period.

Bivariate and multivariate logistic regression analysis was performed to estimate the likelihood for a CS during the final time period in comparison with the first, adjusting for maternal age, referral status, and private healthcare insurance. Only those variables of significance in the bivariate analysis were included in the multivariate analysis. Data was presented as odds ratios (OR) and 95% confidence intervals (CI). We adjusted for maternal age, referral status, and private healthcare insurance to determine if they could explain the increase in CS rate. High maternal age is a known risk factor for CS [26], and there were reports of increased maternal age at MNH [14]. Being referred by another hospital was considered to increase the risk of CS, as referral status was associated with a higher CS rate in a previous study from Tanzania and might reflect the presence of a complication [20]. Because of the reorganisation of obstetric care during the study period, the proportion of referred patients had increased at MNH [23]. Previous studies also revealed a higher CS rate among women with private healthcare insurance than for women without such insurance [27].

### Ethics approval

Study procedures closely followed approved ethics guidelines for biomedical research involving human subjects, in compliance with the Helsinki Declaration. The research group worked with completely anonymous data. Clearance to conduct the study was obtained from the Ethics Board at Muhimbili University for Health and Allied Sciences on 25 February 2011 (reference number MU/DRP/REC/VOL.1/25). A research permit was given by the Tanzania Commission for Science and Technology on 17 February 2012 (reference number 2012-39-NA-2011-191). Permission to collect data was obtained from the Muhimbili National Hospital administration.

## Results

There were 139,315 deliveries registered in the obstetric database between 2000 and 2011. Of these, 137,094 (98.4%) were included in the analysis. The categories and numbers of deliveries excluded due to missing or questionable data were as follows: birth weight (1,915), mode of delivery (16), perinatal mortality (38), Apgar score at five minutes (223), and maternal mortality (29).

Table 1 summarises the characteristics of those women delivered at MNH during the study period. Mean maternal age increased from 25.2 to 27.9 years (*p* < 0.001) and mean parity rose from 2.2 to 2.3 (*p* < 0.001). When private healthcare insurance was introduced at MNH in 2004, the number of women with such insurance rose steadily reaching 20% in the last time period. The proportion of women referred from other hospitals increased from 7.2% to 28%. The use of vacuum extraction remained at a constant low of 0.8%.

**Table 1:** Characteristics of women delivered at Muhimbili National Hospital, 2000 to 2011

		**2000–2002**	**2003–2005**	**2006–2008**	**2009–2011**	**2000–2011**
Maternal age	Mean (SD ^a^)	25.2 (6.1)	26.0 (5.9)	27.0 (5.8)	27.9 (5.7)	26.3 (6.0)
	Range	12–50	12–50	12–50	13–50	12–50
	Missing (%)	811 (1.7%)	679 (2.0%)	287 (1.0%)	183 (0.6%)	1960 (1.4%)
Parity	Mean (SD)	2.2 (1.6)	2.2 (1.5)	2.1 (1.3)	2.3 (1.4)	2.2 (1.5)
	Range	1–14	1–15	1–13	1–13	1–15
Previous CS ^b^	Previous CS (%)	3365 (7.2%)	2964 (8.6%)	2739 (9.9%)	4859 (17%)	13, 927 (10%)
	No previous CS (%)	43,357 (93%)	31, 416 (91%)	24, 896 (90%)	23, 498 (83%)	123, 167 (90%)
Private insurance	Private (%)	0 (0%)	2268 (6.6%)	5257 (19%)	5702 (20%)	13, 227 (9.6%)
	Public (%)	46,722 (100%)	32,112 (93%)	22,378 (81%)	22,655 (80%)	123, 867 (90%)
Referral status	Referred from another hospital (%)	3351 (7.2%)	3946 (11%)	4897 (18%)	7979 (28%)	20,173 (15%)
	Not referred from another hospital (%)	43,371 (93%)	30,434 (89%)	22,738 (82%)	20,378 (72%)	116,921 (85%)
Instrumental deliveries (%)		337 (0.7%)	384 (1.1%)	133 (0.5%)	302 (1.1%)	1156 (0.8%)

^a^SD = Stardard Deviation.

^b^CS = Caesarean section.

The total CS rate rose from 19% during the first time period to 49% in the last time period (Table 2). The rise was significant for all groups (*p* < 0.001) except transverse lie (*p* = 0.319). Multipara with no previous CS and a single cephalic pregnancy with induced labour or elective CS (group 4) had the highest increase in CS rate (from 26% to 91%). Other groups with high increase in CS rate were women with single cephalic pregnancy with birth weight < 2.5 kg (from 13% to 22%) and multipara with no previous CS and a single cephalic pregnancy in spontaneous labour (from 12% to 33%). During the entire study period, the CS rate was 46% among private patients and 29% among public patients.

**Table 2:** Caesarean section rate (CSs over total number of deliveries) in the ten Robson groups, 2000 to 2011, and relative change between first and last time period

**Robson group**	**2000–2002**	**2003–2005**	**2006–2008**	**2009–2011**	**2000–2011**	**Relative change in %**
1. Nullipara, single, cephalic, birth weight ≥ 2.5 kg, spontaneous labour	17% (2628/15,688)	26% (2709/10,607)	35% (2998/8687)	44% (3250/7430)	27%^a^ (11,585/42,412)	159%^c^
2. Nullipara, single, cephalic, birth weight ≥ 2.5 kg, induced labour or elective CS	37% (87/236)	59% (88/150)	70% (47/67)	91% (290/320)	66%^a^ (512/773)	146%^c^
3. Multipara, no previous CS, single, cephalic, birth weight ≥ 2.5 kg, spontaneous labour	12% (2148/17,679)	19% (2644/14,229)	28% (2974/10,749)	33% (3293/10,098)	21%^a^ (11,059/52,755)	175%^c^
4. Multipara, no previous CS, single, cephalic, birth weight ≥ 2.5 kg, induced labour or elective CS	26% (39/152)	48% (48/101)	74% (32/43)	91% (472/518)	73%^a^ (591/814)	250%^c^
5. Previous CS, single, cephalic, birth weight ≥ 2.5 kg	75% (2213/2948)	85% (2177/2561)	95% (2284/2404)	95% (3991/4182)	88%^a^ (10,665/12,095)	27%^c^
6. Nullipara, single, breech	29% (137/469)	44% (91/208)	58% (104/178)	67% (121/182)	44%^a^ (453/1037)	131%^c^
7. Multipara, single, breech	21% (128/597)	27% (95/354)	38% (91/237)	57% (161/284)	32%^a^ (475/1472)	171%^c^
8. Multiple pregnancies	23% (375/1638)	34% (425/1237)	44% (483/1094)	52% (622/1189)	37%^a^ (1905/5158)	126%^c^
9. Single, transverse or oblique lie	85% (239/282)	93% (160/173)	67% (4/6)	88% (60/68)	88%^b^ (463/529)	3.5%^c^
10. Single, cephalic, birth weight < 2.5 kg	13% (941/7033)	23% (1082/4760)	23% (976/4170)	37% (1494/4086)	22%^a^ (4493/20,049)	185%^c^
Total	19% (8935/46,722)	28% (9519/34,380)	36% (9993/27,635)	49% (13,754/28,357)	31%^a^ (42,201/137,094)	158%^c^

^a^P -value < 0.001 using the *χ*2 test for change.

^b^P -value = 0.319 using the *χ*2 test for change.

^c^(CS rate last time period – CS rate first time period)/CS rate first time period.

There were some changes in the relative size of the Robson groups during the study period (Additional file 1: Table A1). Nullipara with single cephalic pregnancies in spontaneous labour (group 1) and multipara without previous CS with single cephalic pregnancies in spontaneous labour (group 3) decreased in relative size. During the final years, there was a sharp increase in women with previous CS (group 5). Multipara without previous CS with single cephalic pregnancies in spontaneous labour (group 3) constituted the largest group throughout the study period.

The three largest groups (groups 1, 3, and 5) contributed most to the total CS rate over the study period (Additional file 1: Table A2). Nullipara with single cephalic pregnancies in spontaneous labour (group 1) contributed 5.6% in the first time period and 12% in the last time period; multipara with single cephalic pregnancies in spontaneous labour (group 3) contributed 4.6% in the first time period and 12% in the last time period; and multipara with previous scar (group 5) contributed 4.7 % in the first time period and 14% in the last time period. Consequently, multipara with previous scar (group 5) were the largest contributor to the total CS rate in the last time period.

Perinatal and maternal outcomes revealed both improvements and declines. There was a total reduction in the perinatal mortality ratio (Table 3) and the proportion of neonatal distress (Additional file 1: Table A3) (*p* < 0.001 for both). Groups with decreases in both of these variables included nullipara with single cephalic pregnancies in spontaneous labour (group 1), nullipara with induced labour or elective CS (group 2), women with previous CS (group 5), nullipara with breech presentation (group 6), and multiple pregnancies (group 8). Multipara with induced labour or elective CS (group 4), multipara with breech presentation (group 7), and single cephalic pregnancies with birth weight < 2.5 kg (group 10) showed decreased perinatal mortality but either no change or an increase in neonatal distress. Multipara with single cephalic pregnancies in spontaneous labour (group 3) had increased perinatal mortality (from 55 per 1000 deliveries in the first time period to 59 per 1000 deliveries in the last) (*p* = 0.381) and neonatal distress (from 4.1% in the first time period to 4.7% in the last time period) (*p* = 0.171). The maternal mortality ratio increased from 453 per 100, 000 live births in the first time period to 650 per 100, 000 live births in the last time period (*p* = 0.031).

**Table 3:** Perinatal mortality ratio (number of stillbirths and early neonatal deaths per 1000 deliveries) in the ten Robson groups, 2000 to 2011

**Robson group**	**2000–2002**	**2003–2005**	**2006–2008**	**2009–2011**	**2000–2011**	**p -value**
1. Nullipara, single, cephalic, birth weight ≥ 2.5 kg, spontaneous labour	70 (1094/15,688)	55 (584/10,607)	61 (533/8687)	55 (410/7430)	62 (2621/42,412)	< 0.001
2. Nullipara, single, cephalic, birth weight ≥ 2.5 kg, induced labour or elective CS	110 (26/236)	153 (23/150)	119 (8/67)	31 (10/320)	87 (67/773)	< 0.001
3. Multipara, no previous CS, single, cephalic, birth weight ≥ 2.5 kg, spontaneous labour	55 (976/17,679)	50 (713/14,229	52 (556/10,749)	59 (592/10,098)	54 (2837/52,755)	0.381
4. Multipara, no previous CS, single, cephalic, birth weight ≥ 2.5 kg, induced labour or elective CS	86 (13/152)	99 (10/101)	23 (1/43)	46 (24/518)	59 (48/814)	0.023
5. Previous CS, single, cephalic, birth weight ≥ 2.5 kg	43 (126/2948)	27 (68/2561)	36 (86/2404)	29 (121/4182)	33 (401/12,095)	0.014
6. Nullipara, single, breech	299 (140/469)	207 (43/208)	185 (33/178)	165 (30/182)	237 (246/1037)	< 0.001
7. Multipara, single, breech	307 (183/597)	246 (87/354)	241 (57/237)	243 (69/284)	269 (396/1472)	0.024
8. Multiple pregnancies	158 (258/1638)	132 (163/1237)	109 (119/1094)	96 (114/1189)	127 (654/5158)	< 0.001
9. Single, transverse or oblique lie	238 (67/282)	191 (33/173)	167 (1/6)	176 (12/68)	214 (113/529)	0.197
10. Single, cephalic, birth weight < 2.5 kg	334 (2347/7033)	316 (1504/4760)	323 (1345/4170)	302 (1234/4086)	321 (6430/20,049)	0.002
Total	112 (5230/46,722)	94 (3228/34,380)	99 (2739/27,635)	92 (2616/28,357)	101 (13,813/137,094)	< 0.001

P-value for test of trend.

ORs of likelihood of a CS during the final time period in comparison with the first are presented in Table 4. Maternal age, referral status, or private healthcare insurance affected the OR of CS among all Robson groups, except multipara with induction or elective CS (group 4). Adjustment for maternal age or private healthcare insurance independently changed the OR of CS in seven out of ten groups. Referral status affected the OR of CS among half of the groups. For all Robson groups (except group 9), the OR of CS after adjustments remained significantly higher in the last time period compared with the first. For the total study population, the OR of CS in the last time period was 2.8 (CI 2.7 to 2.9) after adjustments.

**Table 4:** Bivariate and multivariate logistic regression analysis of likelihood of CS in the ten Robson groups during last time period compared with first time period

**Robson group**	**2000–2002**	**2009–2011**	
	**OR**	**Crude OR (95% CI)**	**Adjusted OR (95% CI)**
1. Nullipara, single, cephalic, birth weight ≥ 2.5 kg, spontaneous labour	1	3.9 (3.6–4.1)	2.4 (2.2–2.6)^a^
2. Nullipara, single, cephalic, birth weight ≥ 2.5 kg, induced labour or elective CS	1	17 (11–26)	12 (7.5–20)^b^
3. Multipara, no previous CS, single, cephalic, birth weight ≥ 2.5 kg, spontaneous labour	1	3.5 (3.3–3.7)	2.6 (2.4–2.8)^a^
4. Multipara, no previous CS, single, cephalic, birth weight ≥ 2.5 kg, induced labour or elective CS	1	30 (19–48)	30 (19–48)^c^
5. Previous CS, single, cephalic, birth weight ≥ 2.5 kg	1	6.9 (5.9–8.2)	6.3 (5.1–7.8)^d^
6. Nullipara, single, breech	1	4.8 (3.3–6.9)	2.6 (1.7–4.0)^a^
7. Multipara, single, breech	1	4.8 (3.5–6.5)	4.1 (3.0–5.7)^e^
8. Multiple pregnancies	1	3.7 (3.1–4.3)	3.1 (2.6–3.7)^f^
9. Single, transverse or oblique lie	1	1.4 (0.6–3.0)	1.4 (0.6-3.0)^c^
10. Single, cephalic, birth weight < 2.5 kg	1	3.7 (3.4–4.1)	2.7 (2.5–3.1)^a^
Total	1	4.0 (3.9–4.1)	2.8 (2.7–2.9)^a^

^a^adjusted for maternal age, referral status, and private healthcare insurance.

^b^adjusted for maternal age, no significant change from referral status or private healthcare insurance.

^c^no significant change from any of the factors.

^d^adjusted for maternal age and private healthcare insurance, no significant change from referral status.

^e^adjusted for private healthcare insurance, no significant change from maternal age or referral status.

^f^adjusted for referral status and private healthcare insurance, no significant change from maternal age.

Odds ratios (OR) and 95% confidence intervals (CI).

## Discussion

We found a sharp increase in the total CS rate for all obstetric groups except transverse lie. The group of women with previous CS (group 5) increased its relative size during the study period and was the main contributor to the total CS rate in the last time period. Although there was an overall improvement in perinatal outcomes, it did not include all groups. The largest group, women without previous CS with single cephalic pregnancies in spontaneous labour, had no improved perinatal outcomes and a remarkably high CS rate. In total, maternal mortality increased during the study period. Maternal age, referral status, and private healthcare insurance affected the CS rate in most groups, but the OR remained high for CS in the last time period after adjusting for these factors.

The study incorporated a large sample size over a long time span. Although some deliveries were excluded due to missing data, we do not believe this affected the results since those deliveries only accounted for 1.6% of the total. However, as in many other register studies, there might have been an underreporting in some variables. For example, group 9 (transverse lie) presented large differences in the total number of pregnancies between the years, indicating that this presentation was underreported during some time periods. A previous CS could have been missed, leading to a woman being classified into the wrong Robson group. The relative size of the groups with induction and elective CS (groups 2 and 4) were very small in comparison with other studies [18-20]. This might be partly explained by a local tradition of rarely performing induction or elective CS, but might also result from an underreporting of induction. Also, information was usually lacking about socioeconomic status, maternal weight, and complications due to other diseases, all of which would have been valuable when interpreting the results.

Another limitation was the development of the database, as new variables were introduced and others modified during the study period. An example of this was the redefinition of gestational age, for which we used birth weight to distinguish term and preterm deliveries. Birth weight < 2.5 kg is defined as low by the World Health Organization, including both preterm and growth-restricted newborns. In our dataset, the percentage of babies with this birth weight was in accordance with the estimated prevalence of preterm birth in sub-Saharan Africa [28]. Thus, despite some limitations, we believe our study provides useful information on time trends with regard to CS and outcomes.

Although adjusted analysis explained some of the increase in CS rate, the high CS rate in low-risk groups, such as multipara without previous CS with single cephalic pregnancies (group 3), implies that many CSs might have been done on questionable indications. This finding is in agreement with studies from high- and middle-income countries where rising CS rates have not been explained by increased risk indication [7,9] and another study from northern Tanzania that revealed many CSs done on questionable indications [16]. Private practice and economic incentives have been reported to affect CS decision making at other institutions [8,10], and might also play a role at MNH as the CS rate was remarkably higher among private patients. CSs performed on non-medical indications are no longer only an issue for high- and middle-income countries. There is a risk that excess use of this procedure, especially in settings with low resources, might act as a barrier to other, more effective improvements in obstetric care [6]. Also, doctors trained at a teaching hospital with high CS rates might continue this practice in their future career.

As a result of the shift in obstetric practice from vaginal to caesarean birth, the proportion of women with previous CS had increased substantially. In the last time period, the relative size of the group was comparable to studies from high- and middle-income countries [18-20] and women with previous CS contributed most to the total CS rate. The CS rate for women with previous CS usually ranges from 51% to 83% in other studies [18-20], but was found to be 95% in our study. We interpret the high rate of repeat CS in our study as a cautious attitude on the part of the staff at MNH. There is no apparatus for cardiotocography to monitor labour and each midwife attends several birthing women simultaneously. Also, the time interval between a CS decision and operation is usually long. Therefore, obstetricians at MNH may elect to perform an early CS than risk a uterine rupture that might jeopardize the life of the mother and the baby. Considering the increasing proportion of women with previous CS and the high CS rate in Group 5, the total CS rate is likely to increase in coming years.

We believe the present study is the first to compare outcomes and CS rates in the ten Robson groups over time. Although we cannot conclude that the changes in outcomes we found were a result of the increase in the CS rate, the improvement in total perinatal outcomes suggests that the increasing CS rate might have had a positive effect on perinatal health. However, the largest obstetric group, multipara without previous scar (group 3), had no improved perinatal outcomes despite increased CS rates. Another study from Nigeria also reported a lack of improvement in perinatal outcomes despite increased CS rates [9]. We conclude that while CS can improve perinatal outcomes, it does not necessarily do so if performed routinely in a low-risk group. Also, the significant increase in maternal mortality ratio revealed by our study is alarming and calls for attention to the effect caesarean delivery might have on maternal health in a low-income country.

We found the Robson classification to be readily applicable in a low-resource setting. In our study, we used birth weight instead of gestational age. As most women in low-income countries do not undergo ultrasound during pregnancy and accurate data on gestational age is difficult to obtain [28], this approach may allow the Robson classification to be used in a low-resource setting. Since the Robson classification only requires basic data collected in most delivery wards, it can be implemented at any facility to monitor and evaluate CS rates. By doing this prospectively with continuous feed-back to the staff, the quality of obstetric care may be improved.

Results from this study may be generalised and implemented to other referral institutions in low-income countries with high CS rates. In many hospitals, high CS rates are accompanied by a low usage of instrumental delivery [15,29]. At MNH, the high CS rate among nullipara and multipara without previous CS with a single cephalic pregnancy in spontaneous labour (groups 1 and 3) illustrates this situation. Potential interventions to lower the CS rate and improve outcomes may be increasing the use of vacuum extraction, auditing CS decisions and the use of partograms, introduce a mandatory second opinion for CS decision making, and promoting active management of labour. The aforementioned have proven effective elsewhere [30]. Moreover, the high rate of repeat caesareans among women with previous CS calls for improvements in the organization, such as a more structured surveillance during labour and a shortening of the time interval between CS decision and operation, in order to allow women with previous CS to have a trial of scar.

## Conclusion

Our study has sought a better understanding of the increasing CS rate at a university hospital in a low-income country by identifying a high CS rate among obstetric low-risk groups. Because the high CS rate among low-risk groups suggests that many CSs were done on questionable indications, further studies should focus on healthcare-related factors behind the rising CS rate by interviewing caregivers. Our results can be used to plan interventions to lower CS rates and improve outcomes at this and similar facilities.

## Competing interests

The authors declare that they have no competing interests.

## Authors’ contributions

HL, HK, ED, and BE planned the study. HL, HK, and LN conducted the data analysis. All authors contributed to the interpretation of the results with their critical comments and assisted in revising the manuscript. All authors read and approved the final manuscript.

## Pre-publication history

The pre-publication history for this paper can be accessed here:

http://www.biomedcentral.com/1471-2393/13/107/prepub

## Supplementary Material

Additional file 1 **Table A1.** Relative size of the ten Robson groups, 2000 to 2011. **Table A2**. Absolute contribution of the ten Robson groups to the total caesarean section rate, 2000 to 2011. **Table A3**. Neonatal distress in the ten Robson groups, 2000 to 2011. Click here for file
