# Supplementary material for: Increasing caesarean section rates among low-risk groups: a panel study classifying deliveries according to Robson at a university hospital in Tanzania
Source: BMC Pregnancy Childbirth. 2013 May 8;13:107. doi: 10.1186/1471-2393-13-107 (PMC3655870; doi:10.1186/1471-2393-13-107)
Supplement: Supplementary file 1 [file 1471-2393-13-107-S1.doc]

Table A1. Relative size (deliveries within group over total number of deliveries) of the ten Robson groups, 2000 to 2011. *P* -value for test of trend.

Robson group	2000–2002	2003–2005	2006–2008	2009–2011	2000–2011	*p* -value
1. Nullipara, single, cephalic, birth weight > 2.5 kg, spontaneous labour	33.6%	30.9%	31.4%	26.2%	30.9%	< 0.001
2. Nullipara, single, cephalic, birth weight > 2.5 kg, induced labour or elective CS	0.5%	0.4%	0.2%	1.1%	0.6 %	< 0.001
3. Multipara, no previous CS, single, cephalic, birth weight > 2.5 kg, spontaneous labour	37.8%	41.4%	38.9%	35.6%	38.5%	< 0.001
4. Multipara, no previous CS, single, cephalic, birth weight > 2.5 kg, induced labour or elective CS	0.3%	0.3%	0.2%	1.8%	0.6%	< 0.001
5. Previous CS, single, cephalic, birth weight > 2.5 kg	6.3%	7.4%	8.7%	14.7%	8.8%	< 0.001
6. Nullipara, single, breech	1.0%	0.6%	0.6%	0.6%	0.8%	< 0.001
7. Multipara, single, breech	1.3%	1.0%	0.9%	1.0%	1.1%	< 0.001
8. Multiple pregnancies	3.5%	3.6%	4.0%	4.2%	3.8%	< 0.001
9. Single, transverse or oblique lie	0.6%	0.5%	0.0%	0.2%	0.4%	< 0.001
10. Single, cephalic, birth weight < 2.5 kg	15.1%	13.8%	15.1%	14.4%	14.6%	0.19
Total	100%	100%	100%	100%	100%	

Table A2. Absolute contribution (number of CS in the group/total number of deliveries during the epoch) of the ten Robson groups to the total caesarean section rate, 2000 to 2011.

Robson group	2000-2002	2003-2005	2006-2008	2009-2011	2000-2011
1. Nullipara, single, cephalic, birth weight > 2.5 kg, spontaneous labour	5.6%	7.9%	11%	12%	8.5%
2. Nullipara, single, cephalic, birth weight > 2.5 kg, induced labour or elective CS	0.2%	0.3%	0.2%	1.0%	0.4%
3. Multipara, no previous CS, single, cephalic, birth weight > 2.5 kg, spontaneous labour	4.6%	7.7%	11%	12%	8.1%
4. Multipara, no previous CS, single, cephalic, birth weight > 2.5 kg, induced labour or elective CS	0.1%	0.1%	0.1%	1.7%	0.4%
5. Previous CS, single, cephalic, birth weight > 2.5 kg	4.7%	6.3%	8.3%	14%	7.8%
6. Nullipara, single, breech	0.3%	0.3%	0.4%	0.4%	0.3%
7. Multipara, single, breech	0.3%	0.3%	0.3%	0.6%	0.3%
8. Multiple pregnancies	0.8%	1.2%	1.7 %	2.2%	1.4%
9. Single, transverse or oblique lie	0.5%	0.5%	0%	0.2%	0.3%
10. Single, cephalic, birth weight < 2.5 kg	2.0%	3.1%	3.5%	5.3%	3.3%

Table A3. Neontal distress (number of live births with Apgar score < 7 at 5 minutes per total number of live births) in the ten Robson groups, 2000 to 2011. *P*-value for test of trend.

Robson group	2000–2002	2003–2005	2006–2008	2009–2011	2000–2011	*p*-value
1. Nullipara, single, cephalic, birth weight > 2.5 kg, spontaneous labour	9.8% (1466/14,942)	8.3 % (849/10,181)	8.0% (670/8393)	8.3% (600/7192)	8.8% (3585/40,708)	< 0.001
2. Nullipara, single, cephalic, birth weight > 2.5 kg, induced labour or elective CS	23% (49/211)	17.2% (22/128)	16.4% (10/61)	8.6% (27/314)	15.1% (108/714)	< 0.001
3. Multipara, no previous CS, single, cephalic, birth weight > 2.5 kg, spontaneous labour	4.1% (698/16,858)	4.2% (570/13,619)	3.9% (404/10,317)	4.7% (449/9634)	4.2% (2121/50,428)	0.171
4. Multipara, no previous CS, single, cephalic, birth weight > 2.5 kg, induced labour or elective CS	7.1% (10/141)	4.3% (4/92)	11.6% (5/43)	6.3% (32/511)	6.5% (51/787)	0.916
5. Previous CS, single, cephalic, birth weight > 2.5 kg	5.8% (164/2843)	3.7% (93/2503)	4.1% (96/2356)	3.2% (129/4087)	4.1% (482/11,789)	< 0.001
6. Nullipara, single, breech	18.5% (66/356)	12.8% (22/172)	11.7% (18/154)	12.0% (19/158)	14.9% (125/840)	0.024
7. Multipara, single, breech	15.4% (69/447)	18.5% (53/286)	19.7% (38/193)	17.7% (41/232)	17.4% (201/1158)	0.326
8. Multiple pregnancies	10.4% (154/1487)	10.0% (114/1135)	8.1% (83/1025)	7.3% (82/1124)	9.1% (433/4771)	0.003
9. Single, transverse or oblique lie	17.6% (40/227)	13.4% (19/142)	0% (0/5)	22.8% (13/57)	16.7% (72/431)	0.603
10. Single, cephalic, birth weight < 2.5 kg	17.5% (903/5155)	19.6% (690/3528)	20.8% (648/3122)	20.6% (646/3138)	19.3% (2887/14,943)	< 0.001
Total	8.5% (3619/42,667)	7.7% (2436/31,786)	7.7% (1972/25,669)	7.7% (2038/26,447)	8.0% (10,065/126,569)	< 0.001
